# Functional Audiometric Dissociation in Ménière’s Disease: Exploring the Mismatch Between Pure-Tone Thresholds and Speech Recognition

**DOI:** 10.3390/jcm14134747

**Published:** 2025-07-04

**Authors:** Joan Lorente-Piera, Melissa Blanco, Javier Santos-Garrido, Raquel Manrique-Huarte, Víctor Suárez-Vega, Pablo Domínguez, Jaime Fullaondo, Lorea Arbizu, Nicolás Pérez-Fernández

**Affiliations:** 1 Department of Otorhinolaryngology, Clínica Universidad de Navarra, 31001 Pamplona, Spain; joanlopi5@gmail.com (J.L.-P.); rrmanriqeu@unav.es (R.M.-H.); 2 Department of Otorhinolaryngology, Clínica Universidad de Navarra, 28001 Madrid, Spain; mblancop@unav.es (M.B.); nperezfer@unav.es (N.P.-F.); 3 Department of Radiology, Clínica Universidad de Navarra, 28001 Madrid, Spain; 4 Department of Radiology, Clínica Universidad de Navarra, 31001 Pamplona, Spain; 5 Centro Médico de Asturias, 33193 Oviedo, Spain; 6 Department of Otorhinolaryngology Complejo Hospitalario Universitario de Navarra, 31001 Pamplona, Spain

**Keywords:** Ménière’s disease, hearing loss, endolymphatic hydrops, perilymphatic enhancement

## Abstract

**Background/Objectives**: Tonal thresholds, typically assessed through pure-tone audiometry (PTA), are central to the audiological evaluation of Ménière’s disease (MD). However, they fail to capture the complexity of real-life speech perception. This study aimed to characterize the relationship between PTA and speech recognition performance in unilateral MD and to determine whether a measurable dissociation exists between hearing sensitivity and verbal processing. We also evaluated frequency-specific audiometric patterns and potential threshold cut-off values associated with speech recognition decline. **Methods**: A total of 254 ears from 127 patients were included in the study across three groups: the Ménière group (affected and unaffected ears, n = 64 each) and the control group (n = 63). The pure-tone thresholds, speech recognition threshold (SRT), and the maximum word recognition scores (Rmax) were assessed in all participants. **Results:** Although the Ménière affected and control groups showed comparable pure-tone average (PTA) values (46.33 vs. 38.13 dB, *p* = 0.439), the affected group demonstrated significantly poorer speech performance (Rmax: 76.25% vs. 87.49%, *p* < 0.001; SRT: 50.64 vs. 38.45 dB, *p* = 0.009). The Ménière unaffected group exhibited near-ceiling performance (Rmax: 99.38%, SRT: 18.33 dB) and a mean PTA of 19.59 dB. A strong correlation between PTA and SRT was observed only in the Ménière affected group (r = 0.942, *p* < 0.001), whereas correlations were moderate in the unaffected (r = 0.671, *p* < 0.001) and control (r = 0.728, *p* < 0.001) groups. The ROC analysis revealed that PTA predicted impaired speech recognition with high accuracy in unaffected (AUC = 0.956, *p* < 0.001) and control (AUC = 0.829, *p* < 0.001) ears but far less so in affected ears (AUC = 0.784; all *p* < 0.001), confirming a functional tonal–verbal dissociation in MD. **Conclusions**: This study demonstrates a functional audiometric dissociation in unilateral Ménière’s disease. Affected ears show poorer speech recognition and require higher intensities despite similar PTA values. The predictive link between thresholds and verbal performance is disrupted. These findings support the need for combined tonal and speech-based assessment in clinical practice.

## 1. Introduction

Ménière’s disease (MD) is a chronic inner ear disorder characterized by recurrent episodes of vertigo, fluctuating sensorineural hearing loss (SNHL), tinnitus, and aural fullness [1]. Idiopathic endolymphatic hydrops is considered the primary histopathological correlation of the disease, and it leads to a variable degree of cochlear and vestibular dysfunction [2,3]. While vertigo is typically the most disabling symptom, hearing loss significantly impairs patients’ quality of life and functional communication.

Pure-tone audiometry has traditionally been used to monitor auditory thresholds in MD, and it remains the cornerstone for diagnostic classification and staging [4]. In the early stages, the hearing loss typically affects the low frequencies and follows a fluctuating course. As the disease progresses, audiometric thresholds tend to worsen across all frequencies. This leads to either a flat or progressively sloping audiometric configuration. In some cases, the hearing loss may reach profound levels, with approximately 6% of patients eventually becoming candidates for cochlear implantation [5,6].

Although the shape of the audiogram is not directly dependent on disease duration, early deterioration—particularly involving mid- and high-frequency thresholds—has been associated with a poorer auditory prognosis. Another important aspect to consider when evaluating hearing loss in patients with Ménière’s disease relates to the onset pattern. Although the classical triad of symptoms is well known, most patients do not present with all three simultaneously. In fact, atypical presentations—where the disease begins with one or two symptoms—are more common. When the disease starts with bisymptomatic onset, the most common combination (19.8%) involves cochlear symptoms, specifically hearing loss and tinnitus. The time from the onset of the first symptom until the full triad develops varies considerably. In this type of debut onset, only with cochlear symptoms, the median time to completion of the symptom triad is approximately 2 years, with the 25th and 75th percentiles at 0.74 and 4.9 years, respectively [7]. Nevertheless, these diagnostic and prognostic frameworks tend to emphasize pure-tone audiometry, while the assessment of speech recognition remains underexplored and often undervalued in clinical practice.

Despite the emphasis on PTA in clinical practice and research, less attention has been paid to speech recognition thresholds (SRTs) and their progression or fluctuation in MD. In particular, tonal–verbal dissociation remains an underexplored relationship; existing evidence suggests that while some individual cases may exhibit disproportionately poor speech recognition relative to their PTA, these instances are limited and do not represent a generalizable trend [8,9]. Previous studies by our group, based on a prospective follow-up of approximately 80 months, indicated that this dissociation tends to increase over time and shows a statistically significant association with disease duration [10]. This indicates that speech recognition or discrimination during daily activities worsens significantly over pure-tone hearing deterioration. Moreover, patients with MD may experience fluctuations in speech recognition without corresponding changes in PTA throughout follow-up. It is important to note, however, that this prior work had a methodological limitation: speech recognition was assessed at a fixed intensity of 65 dB HL, which for some participants could be less than the required intensity to achieve their maximum word recognition score (Rmax). This is an aspect that may limit the representativeness of the results in real-world listening conditions.

In this context, Rmax, measured by speech material at 35 dB HL above the SRT, provides a valuable yet underutilized metric for assessing auditory function [11,12,13]. Rmax reflects a patient’s capacity to process complex auditory stimuli and can uncover subtle deficits not captured by pure-tone audiometry alone. In patients with functional dissociation, this score may be significantly reduced despite only moderate PTA impairment, revealing inefficiencies in auditory processing or impaired cochlear dynamics. While some individuals with elevated PTA maintain good speech recognition scores at expected intensity levels, others require much higher input or fail to reach satisfactory verbal performance—underscoring the need to systematically assess both tonal thresholds and speech-processing abilities to fully characterize auditory dysfunction.

The objective of this study was to explore the relationship between pure-tone thresholds and speech recognition performance in patients with unilateral MD, with a particular focus on identifying the presence of a functional audiometric dissociation. Specifically, we aimed to determine whether patients with similar tonal thresholds exhibit divergent speech comprehension abilities and whether this mismatch can be systematically quantified. Additionally, we examined frequency-specific audiometric patterns and speech-tone mismatches to evaluate their potential as functional markers for diagnosis and monitoring.

## 2. Materials and Methods

### 2.1. Population, Inclusion, and Exclusion Criteria

All patients included provided explicit consent for the use of their data for research purposes, and written informed consent was obtained from all subjects.

The patients in this study were diagnosed of certain Ménière’s disease [14] and underwent a complete neurotologic examination. They were classified in the following phenotypic variants: idiopathic, delayed, familiar, autoimmune, or with migraine [15].

A total of three groups were established for comparison. The inclusion criteria were as follows:

- The first group (Ménière affected group) consisted of the ears clinically affected in patients with a definitive diagnosis of unilateral MD [14].
- These results were compared to those of a second group (Ménière unaffected group), which included the contralateral, clinically unaffected ears of the same patients.
- Finally, a third group (Control group) comprised individuals diagnosed with other otological conditions involving sensorineural hearing loss not related to endolymphatic hydrops.

Exclusion criteria for all groups included the following: (1) a history of ear surgery; (2) other inner ear disorders causing fluctuating hearing loss; (3) a lack of sufficient audiometric data to calculate PTA; and (4) missing Rmax values or values not obtained at the SRT level (dB) + 35 dB.

### 2.2. Clinical Examination and Audiovestibular Assessment

All participants underwent a comprehensive otological and neurotological examination, which included otoscopy and evaluation using videonystagmography (VNG; VisualEyes™ 525, Interacoustics VF505m, Assens, Denmark). The audiovestibular test battery, which is the purpose of this study, comprised pure-tone audiometry (PTA) and speech recognition testing (AC40, Interacoustics, Assens, Denmark).

- The audiological evaluation was as follows: PTA thresholds were measured across frequencies ranging from 250 Hz to 4000 Hz and reported in decibels hearing level (dB HL). Speech recognition scores (SRS) were obtained using the disyllabic word test developed by Cárdenas and Marrero [16].
- As magnetic resonance imaging (MRI) using hydrops-sensitive sequences is required for the diagnosis of certain MD [14], all included patients underwent inner ear imaging accordingly. Scans were conducted using 3 Tesla systems—either the Magnetom Vida or the Magnetom Skyra models (Siemens Healthineers, Erlangen, Germany)—equipped with 20- or 32-channel phased-array coils. Intravenous gadobutrol (Gadovist 0.1 mmol/mL; Bayer AG, Zurich, Switzerland) was administered at a standard dose of 0.1 mL per kilogram of body weight. The image acquisition was performed four hours after contrast administration. The specific MRI protocol used in this study has been previously outlined in detail [17].

### 2.3. Clinical and Audiological Variables

Key variables for auditory performance comparison included PTA measured in decibels (AC 40, Interacoustics AS, Assens, Denmark). Speech intelligibility was evaluated through two parameters: the SRT and the maximum word recognition score (Rmax), which was determined by delivering speech stimuli at an intensity corresponding to SRT plus 35 dB HL. For both MD groups, audiological variables were defined based on the pure-tone audiogram performed closest in time to the MRI scan in which endolymphatic hydrops was identified. In contrast, for the control group, audiometric data were collected from the audiogram closest to the date of the initial hearing rehabilitation consultation, if applicable, or from the most recent follow-up visit in cases where no hearing intervention was required. In this study, functional dissociation was defined as a clinically relevant mismatch between pure-tone thresholds and speech recognition performance. To explore the clinical profile of patients with disproportionate speech deficits relative to tonal sensitivity, we performed a post hoc exploratory classification. Based on the empirical distribution within the Ménière cohort, we defined functional dissociation as either a PTA better than SRT by more than 30 dB HL, or an Rmax more than 10 units lower than the expected value for that PTA. This threshold corresponded to the 75th percentile of the PTA–Rmax difference distribution in our sample and was used solely for descriptive purposes.

In addition to the objective audiovestibular evaluations described above, several complementary variables were analyzed, including demographic data, overall disease duration, duration of hearing loss, and the number of vertigo episodes experienced within the six months preceding the MRI scan in which endolymphatic hydrops was confirmed. This time frame was selected to approximate the temporal window used for audiometric data collection, ensuring consistency across clinical measures.

### 2.4. Statistical Analysis

Descriptive statistics were calculated for all clinical, demographic, and audiological variables across the three study groups: affected ears in Ménière’s disease, unaffected contralateral ears, and sensorineural hearing loss controls. The distribution of continuous variables was assessed using the Shapiro–Wilk test to determine the suitability of parametric versus non-parametric statistical methods.

Pure-tone thresholds were analyzed by frequency (250 Hz to 4000 Hz), comparing mean values across the three groups using one-way ANOVA or Kruskal–Wallis tests, depending on normality. Post-hoc pairwise comparisons were performed using Bonferroni-adjusted *t*-tests or Mann–Whitney U tests as appropriate.

For speech audiometry, both the Rmax and SRT were compared across groups using the same approach. The impact of clinical and demographic variables (age, sex, disease duration, hearing loss duration, and number of vertigo episodes in the 6 months prior to MRI) on PTA and SRT was examined using univariate analyses (Mann–Whitney U or Kruskal–Wallis tests).

The relationship between tonal thresholds and speech recognition performance was assessed within each group using Spearman’s rank correlation. To determine the PTA value at which functional dissociation becomes evident, receiver operating characteristic (ROC) curve analyses were performed using PTA as the predictor of elevated SRT. The area under the curve (AUC), sensitivity, specificity, and optimal cut-off values were calculated based on the maximum Youden index.

A significance level of *p* < 0.05 was used for all statistical tests. Statistical analyses were performed using GraphPad Prism software (v8.0.1, GraphPad Software Inc., San Diego, CA, USA).

## 3. Results

### 3.1. Patients and Overall Results

A total of 254 ears from 127 patients were included in the study. Of the total, 55.11% were women (n = 70), and 44.88% were men (n = 57). The mean age of the patients was 62.39 ± 15.28 years. The average duration of hearing loss from onset was 5.42 ± 9.31 years. From an audiometric standpoint, the mean PTA at the time of diagnosis was 32.19 ± 28.64 dB, while the mean Rmax was 87.25 ± 21.90%, and mean SRT was 35.16 ± 25.76 Db.

### 3.2. Ménière’s Disease Group

A total of 64 patients were included in this group. Based on this, two subgroups were defined: the Ménière affected group, corresponding to the clinically involved ear, and the Ménière unaffected group, based on data from the contralateral, unaffected ear.

A summary of the clinical and demographic characteristics of these patients is presented in Table 1. Statistical significance values are also provided, derived from the Kruskal–Wallis test, to assess the association between these variables and both the PTA and the SRT at the time of data collection, as previously described in Section 2.3. However, none of these variables reached statistical significance.

Among the 64 patients with a diagnosis of definite MD, the distribution of disease onset forms was as follows: 22 patients (34.38%) debuted with the full triad of symptoms, 31.25% (n = 20) debuted only with vertigo, while 20.31% (n = 13) had a bisymptomatic onset with hearing loss and tinnitus, and finally, 9 patients (14.06%) made it only with hearing loss. When comparing auditory outcomes across these subgroups, no statistically significant differences were found for PTA values (*p* = 0.287). However, a significant association was observed between disease onset and SRT values (*p* = 0.003). On the other hand, we found that the most common pattern of hearing loss onset was fluctuating, observed in 54.69% of cases (n = 35), followed by sudden onset (n = 21, 32.81%) and progressive onset (n = 8, 12.50%). Although patients diagnosed with MD who presented with sudden hearing loss showed the poorest auditory outcomes—both in terms of PTA (57.48 ± 29.16 dB) and SRT (56.24 ± 26.18%)—these differences were not statistically significant when compared to the other onset patterns, neither for PTA (*p* = 0.255) nor for SRT (*p* = 0.111).

### 3.3. Control Group

63 patients were included in the control group, all of whom were classified into six categories based on the underlying etiology of their bilateral sensorineural hearing loss. The majority of cases were attributed to presbycusis, comprising 45 patients (71.42%). This was followed by patients with a history of labyrinthitis (n = 8, 12.70%) and those with idiopathic hearing loss (n = 6, 9.52%). Additionally, cases secondary to head trauma and sudden sensorineural hearing loss each accounted for 4 patients (6.35%).

A summary of the clinical and demographic characteristics of the control group is presented in Table 2. Statistical significance values, derived from the Kruskal–Wallis test, were calculated to assess the association of each variable with both the PTA and SRT. Among the variables analyzed, age was the only factor significantly associated with worse outcomes in both PTA (*p* < 0.001) and SRT (*p* < 0.001). In contrast, sex, hearing loss duration, and the mode of hearing loss onset did not reach statistical significance in relation to either audiometric outcome, although hearing loss duration showed a trend toward significance given the proximity of its *p*-values to the 0.05 threshold.

### 3.4. Comparison Between Groups

**Figure 1:**
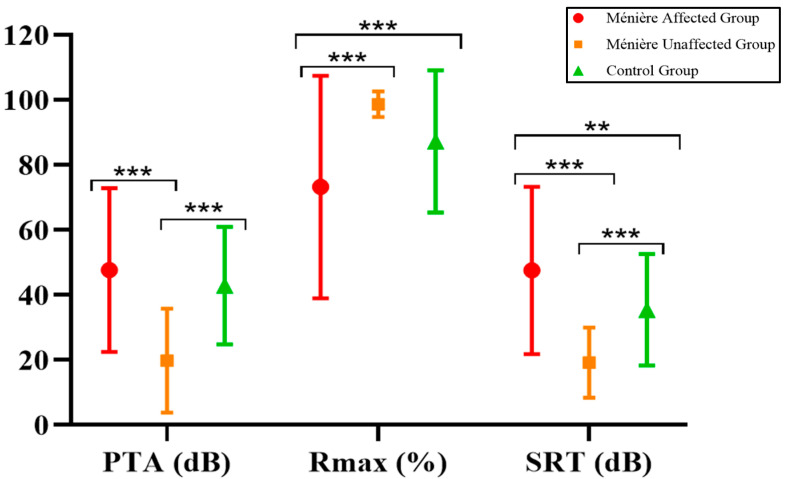
Comparison of overall pure-tone and speech audiometry results in the patients included. ** *p* < 0.01 and *** *p* < 0.001.

When analyzing the results in terms of PTA, Rmax, and SRT, we found that the poorest auditory performance—both in pure-tone thresholds and speech recognition—was observed in the Ménière affected group, followed by the control group and finally the Ménière unaffected group. A comparative analysis using the Mann–Whitney U test revealed several statistically significant differences between groups, as summarized in Table 3 and illustrated in Figure 1.

**Figure 2:**
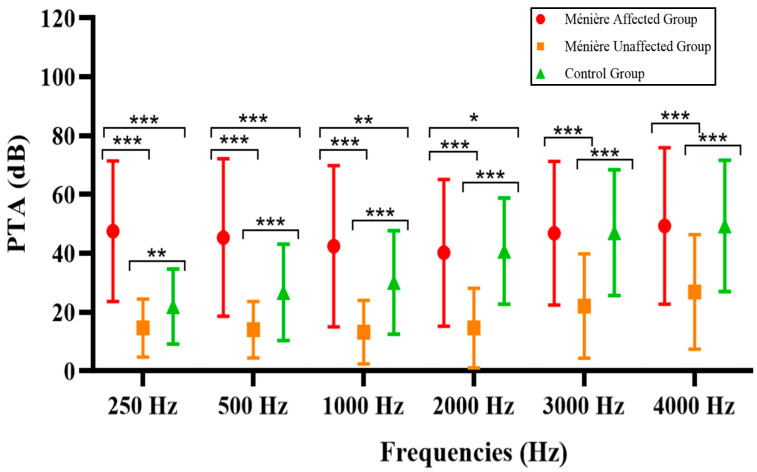
Frequency-specific comparison of the patients in the study across each group. * *p* < 0.05, ** *p* < 0.01, and *** *p* < 0.001.

Of particular interest is the frequency-specific analysis, considering the nature of the diseases included in our study. As outlined in Section 2.3, low, mid, and high frequencies were examined for each subgroup within our cohort, and a comparative analysis was conducted accordingly. The results are presented in Table 4 and Figure 2.

Audiometric comparisons across frequencies revealed, as expected, that the most significant differences between groups were observed at low frequencies (250–1000 Hz), particularly between the Ménière affected group and both the unaffected and control groups. As the frequency increased, the magnitude and statistical significance of the differences progressively diminished.

### 3.5. Relationship Between Pure-Tone Thresholds and Speech Recognition Performance Across Subgroups

**Figure 3:**
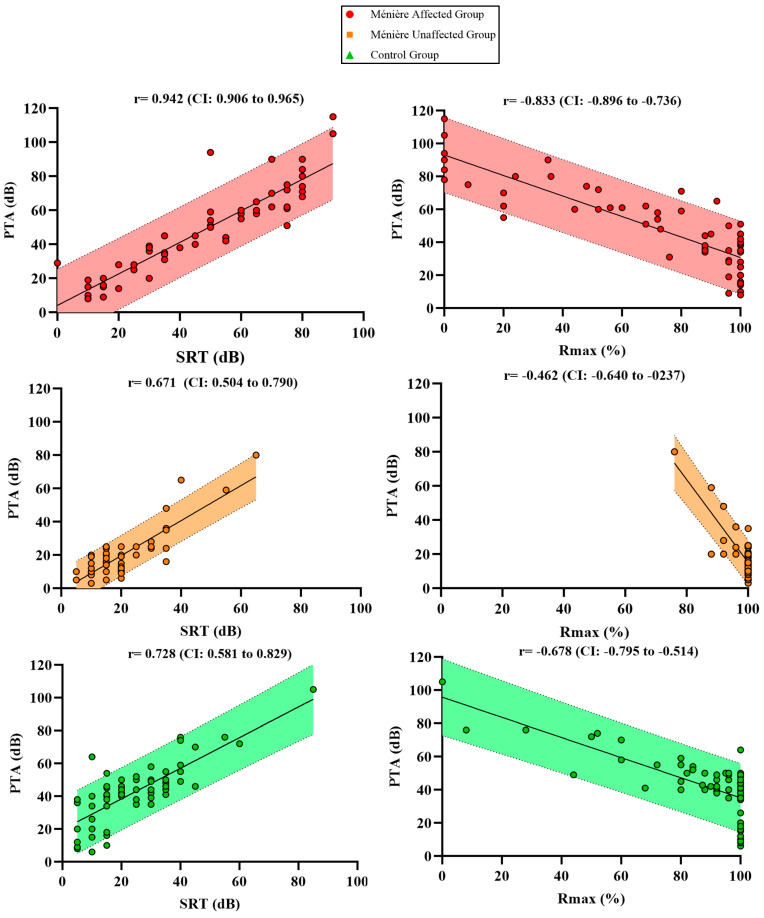
Scatter plots showing the correlations between PTA and SRT as well as PTA + Rmax % across the different groups.

As shown in Figure 3, scatterplots were used to illustrate the relationship between PTA, SRT, and Rmax across the three study groups. Each plot displays the Spearman’s correlation coefficient (r) with its 95% confidence interval, and the shaded area represents the 95% confidence band of the linear regression.

Although a strong correlation was observed within the Ménière affected group (r = 0.942), PTA values were associated with disproportionately elevated SRT levels when compared to the other groups. The Ménière unaffected group showed a moderate correlation (r = 0.671) and the control group a slightly higher one (r = 0.728), both with narrower and more symmetrical distributions. All correlations were statistically significant with *p*-values < 0.001.

To further investigate the tonal–verbal relationship, the correlation between PTA and Rmax was also examined. The Ménière affected group showed a strong negative correlation (r = –0.833), yet many patients exhibited markedly reduced Rmax values even at moderate PTA levels, suggesting an additional layer of functional impairment. The control group displayed a moderate correlation (r = –0.679), where verbal performance declined progressively as hearing loss increased, while the Ménière unaffected group showed a weak correlation (r = –0.462), with Rmax values remaining consistently high despite variability in PTA. Again, all correlations were statistically significant (*p* < 0.001).

These findings suggest that, despite preserved intra-group correlation, the functional relationship between tonal thresholds and verbal performance is altered in affected ears. Not only do Ménière patients require greater intensity to reach maximum intelligibility (SRT), but they also achieve lower peak speech recognition scores (Rmax) compared to other groups with similar PTA values.

To further explore the relationship between tonal and verbal performance, ROC curve analyses were conducted to assess the ability of PTA to predict impaired speech recognition. Separate curves were generated for each of the three study groups: Ménière affected (red), Ménière unaffected (orange), and control (green).

The Ménière unaffected group exhibited the highest discriminative performance, with an AUC of 0.956 (95% CI: 0.910–1.000), indicating an excellent ability of PTA to predict elevated SRT. The control group followed with an AUC of 0.829 (95% CI: 0.760–0.898), consistent with good predictive capacity. In contrast, the Ménière affected group showed a lower AUC of 0.784 (95% CI: 0.704–0.865), revealing a marked decline in the ability of PTA to correctly identify patients with impaired speech recognition in this subgroup.

**Figure 4:**
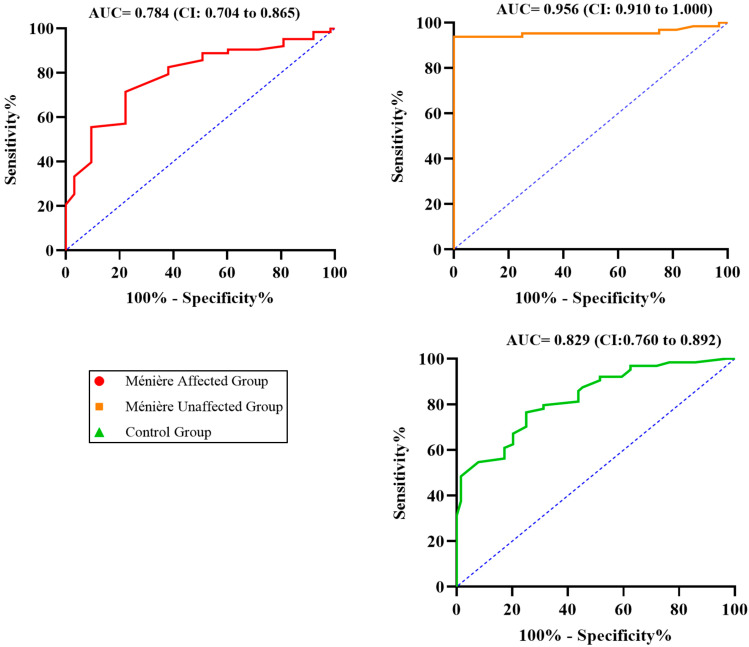
ROC curves evaluating the ability of PTA to predict elevated SRT across the three study groups. The dashed line represents the line of no discrimination (AUC = 0.5).

These results suggest that although PTA values increase alongside SRT in affected ears—as demonstrated by the strong correlation—they lose their discriminative power. In other words, while PTA and SRT remain associated, the ability to use tonal thresholds to classify patients according to their speech performance is significantly compromised in the presence of the MD diagnosis. This discrepancy between correlation and classification reinforces the concept of functional audiometric dissociation: patients with similar PTA values exhibit widely divergent speech intelligibility outcomes in SRT, and many require considerably higher input levels to achieve Rmax. Thus, the ROC analysis provides complementary evidence that supports the disrupted functional relationship between hearing sensitivity and speech comprehension in Ménière’s disease. This is illustrated in Figure 4.

## 4. Discussion

The evaluation of hearing loss in MD has traditionally relied on PTA as the main diagnostic and monitoring tool. However, this approach overlooks a key aspect of functional auditory performance: speech comprehension. In the present study, we sought to characterize the relationship between tonal thresholds and speech intelligibility and to determine whether a functional dissociation exists between the two domains in patients with unilateral MD. Our findings confirm this dissociation from multiple statistical perspectives and highlight the limitations of PTA as a standalone measure in the clinical follow-up of Ménière patients.

The mechanisms contributing to this dissociation are likely complex and multifactorial. As Ménière’s disease progresses clinically, the observed disparity between pure-tone thresholds and speech recognition can no longer be attributed solely to cochlear recruitment [18]. The observed dissociation cannot be explained solely by cochlear recruitment. As the disease progresses, additional mechanisms likely contribute—including retrocochlear involvement. Chronic endolymphatic pressure changes, biochemical alterations, and immune/inflammatory factors may disrupt both outer and inner hair cell function, as well as synaptic transmission at the level of inner hair cells and spiral ganglion neurons [19,20,21]. These changes may impair temporal resolution, neural synchrony, and phase-locking, all of which are essential for speech decoding—particularly in noisy environments [22,23,24,25].

Our findings support this hypothesis. In affected ears, patients required significantly higher intensities to achieve maximal speech intelligibility, despite having PTA values comparable to controls. Although we found a strong negative correlation between PTA and Rmax (r = −0.833), several patients exhibited markedly reduced speech recognition even with only moderate threshold shifts—suggesting deeper dysfunction undetected by PTA alone. In contrast, the unaffected ears and those of the control group maintained near-ceiling Rmax values with much weaker PTA correlations. This supports the notion that the typical tonal–verbal relationship is disrupted in affected ears, as further evidenced by wider data dispersion and altered slope patterns in scatterplots, as well as the reduced discriminative performance of PTA in ROC analyses specific to the affected group (Figure 4). While the dissociation thresholds were defined post hoc based on the internal distribution of our cohort, they may serve as a clinically useful framework to identify patients at risk of underrecognized verbal impairment despite relatively preserved tonal sensitivity. Future studies should aim to replicate and validate these criteria in independent datasets and evaluate their prognostic value for auditory rehabilitation.

It is important to note that, in the ROC analysis, we selected elevated SRT as the outcome variable because it represents the earliest functional manifestation of impaired verbal comprehension, even before the maximal recognition score drops. Unlike Rmax, which may remain artificially preserved due to test ceiling effects or cognitive strategies, SRT elevation reflects a more sensitive indicator of speech intelligibility threshold shift, especially relevant in Ménière’s disease.

Although traditionally understood as a disorder restricted to the inner ear, there is growing evidence that Ménière’s disease involves structural damage beyond simple endolymphatic pressure imbalance [26,27,28,29]. In fact, suprathreshold processing alterations and central plasticity are likely contributors to the tonal–verbal dissociation observed in Ménière’s disease [24,25].

Regarding peripheral organ damage, histological studies have revealed a non-uniform pattern of neural degeneration within the cochlea, with preferential involvement of the apical and middle cochlear turns [30]. This spatial distribution suggests that neural elements responsible for low-frequency processing may be affected earlier or more severely [31]. In this context, although frequencies as low as 250 Hz are not routinely included in standard audiometric analyses for scientific reporting, we considered their inclusion essential. Ménière’s disease is characterized by a predominant low-frequency sensorineural hearing loss, making 250 Hz a clinically and pathophysiologically relevant frequency. By incorporating it into our analysis, we aimed to more accurately capture the functional impact of apical cochlear involvement and enhance the sensitivity of our audiometric assessment to early disease-related changes. This approach aligns with the known tonotopic vulnerability observed in histological and radiological studies.

Our own data align with these histological observations. By analyzing speech performance by frequency range, we identified that speech recognition deficits were more pronounced in frequency bands corresponding to apical cochlear regions, despite the relatively better preservation of hearing thresholds in those frequencies. In the affected ears of patients with Ménière’s disease, we observed a disproportionately reduced speech recognition performance in the lower-frequency range, despite PTA being relatively better preserved in those frequencies. In fact, this difference was statistically significant for low frequencies, as shown in Table 4. In addition, hair cell pathology has been consistently documented in Ménière’s disease, particularly involving disorganization and shortening of stereocilia [32,33,34]. Given the critical role of these structures in cochlear amplification and frequency selectivity, their dysfunction can significantly compromise speech perception [22]. Moreover, a reduction in cochlear gain—especially in the apical turns—may impair the encoding of low-frequency, vowel-rich speech components, ultimately reducing intelligibility even when tonal thresholds remain within a moderate range.

On the other hand, with respect to central or retrocochlear mechanisms, our findings indicate that the steeper regression slopes and shifted intercepts observed in the affected ear with Ménière`s disease reflect a system that requires disproportionately higher input for reduced speech output [35]. This inefficiency reflects both impaired cochlear mechanics and broader deficits in auditory processing [36]. Fluctuations in hearing associated with MD may induce maladaptive central plasticity. Repeated episodes of auditory deprivation and instability could lead to maladaptive central changes, including cortical reorganization, which may further compromise the brain’s ability to process speech, especially when peripheral encoding is already degraded, as demonstrated by our group with functional neuroimaging [37].

Taken together, these results reveal that PTA retains some utility in estimating speech comprehension, but its predictive power is significantly diminished in clinically affected ears. Tonal thresholds are no longer reliable surrogates for functional hearing in the presence of endolymphatic hydrops. This reduction in predictive accuracy—demonstrated across multiple independent metrics—constitutes a robust statistical signature of audiometric dissociation.

While our study provides robust evidence of functional dissociation, several limitations should be acknowledged. First, the cross-sectional design restricts not only causal inference but also the ability to monitor the temporal dynamics of dissociation. It remains unclear whether the tonal–verbal gap progresses over time, stabilizes, or fluctuates with hydrops severity or disease activity. To address this, future longitudinal studies with repeated audiometric and speech recognition assessments are warranted.

Second, although Rmax and SRT were measured using disyllabic word lists in a controlled, standardized environment, this approach lacks ecological validity. Real-world communication typically occurs in complex, noisy, and dynamic settings that demand additional cognitive and auditory processing. The future research should therefore incorporate sentence-based materials, competing noise conditions, or objective indices of listening effort to better approximate real-world auditory performance. Finally, while our control group included a range of SNHL etiologies, further subgroup analysis may help identify distinct dissociation patterns in other pathologies beyond Ménière’s disease.

## 5. Conclusions

In summary, this study demonstrates, through a comprehensive and methodologically diverse approach, the existence of a functional audiometric dissociation in patients with unilateral Ménière’s disease. Despite similar PTA values, affected ears exhibit significantly worse speech recognition, require higher intensities to reach maximal intelligibility, and show reduced predictive alignment between threshold and verbal performance. These findings challenge the assumption that audiometric thresholds are sufficient to describe functional hearing loss and emphasize the need for integrated tonal and verbal assessment in both clinical and research contexts. Therefore, including Rmax in routine audiological assessments of MD patients provides valuable insight into real-world speech comprehension, helping to identify patients with disproportionately poor verbal outcomes despite relatively preserved tonal thresholds. This, in turn, may inform treatment decisions, hearing aid programming, and early referral for cochlear implantation evaluation in selected cases.

## Figures and Tables

**Table 1:** Clinical and demographic results of patients included in the Ménière affected group and Ménière unaffected group.

Clinical and Demographic Data		PTA *p*-Value	SRT *p*-Value
N (men: women)	30:34 patients	0.402	0.194
Age (mean ± SD)	54.75 ± 13.56 (30–79 years)	0.146	0.101
Hearing loss duration (mean ± SD)	6.65 ± 2.80 (1–16 years)	0.105	0.126
MD affected side (right:left)	31:33 patients	0.209	0.118
Phenotype (idiopathic: delayed: familiar: migraine: autoimmune)	49:3:2:6:4 patients	0.368	0.293
Number of dizzy spells last six months (mean ± SD)	6.52 ± 7.52 spells	0.215	0.248

**Table 2:** Clinical and demographic results of patients included in the control group. *** *p* < 0.001.

Clinical and Demographic Data		PTA *p*-Value	SRT *p*-Value
N (men: women)	29:34 patients	0.704	0.308
Age (mean ± SD)	70.16 ± 12.88 (18–93 years)	<0.001 ***	<0.001 ***
Hearing loss duration (mean ± SD)	9.39 ± 15.43 (1–82 years)	0.091	0.085
Onset of hearing loss (sudden: progressive: fluctuating)	7:53:3 patients	0.558	0.279

**Table 3:** Summary and comparison of overall pure-tone and speech audiometry results in the patients included in the study. ** *p* < 0.01 and *** *p* < 0.001.

	MDAff	MDUnaff	Control	MDAff vs. MDUnaff	MDAff vs. Control	MDUnaff vs. Control
PTA.	46.33 ± 26.31 dB	19.59 ± 10.12 dB	38.13 ± 19.68 dB	*p* < 0.001 ***	*p* = 0.439	*p* < 0.001 ***
Rmax	76.25 ± 32.24%	99.38 ± 2.63%	87.49 ± 21.56%	*p* < 0.001 ***	*p* < 0.001 ***	*p* = 0.101
SRT	50.64 ± 22.37 dB	18.33 ± 6.67 dB	38.45 ± 17.58 dB	*p* < 0.001 ***	*p* = 0.009 **	*p* < 0.001 ***

**Table 4:** Summary by frequency of PTA results across the different groups, as well as the statistical comparison between them. * *p* < 0.05, ** *p* < 0.01, and *** *p* < 0.001.

	MDAff	MDUnaff	Control	MDAff vs. MDUnaff	MDAff vs. Control	MDUnaff vs. Control
250 Hz	47.58 ± 23.71 dB	14.61 ± 9.73 dB	21.90 ± 12.68 dB	*p* < 0.001 ***	*p* < 0.001 ***	*p* = 0.002 **
500 Hz	45.39 ± 26.54 dB	14.06 ± 9.56 dB	26.75 ± 16.29 dB	*p* < 0.001 ***	*p* < 0.001 ***	*p* < 0.001 ***
1000 Hz	42.42 ± 27.18 dB	13.28 ± 10.72 dB	30.16 ± 17.45 dB	*p* < 0.001 ***	*p* = 0.009 **	*p* < 0.001 ***
2000 Hz	40.23 ± 24.76 dB	14.61 ± 13.41 dB	40.79 ± 17.87 dB	*p* < 0.001 ***	*p*= 0.018 *	*p* < 0.001 ***
3000 Hz	46.85 ± 24.18 dB	22.09 ± 17.63 dB	47.06 ± 21.26 dB	*p* < 0.001 ***	*p* = 0.547	*p* < 0.001 ***
4000 Hz	49.37 ± 26.37 dB	26.88 ± 19.29 dB	49.34 ± 22.07 dB	*p* < 0.001 ***	*p* = 0.507	*p* < 0.001 ***

## Data Availability

Data pertaining to this study can be shared upon request to the corresponding author.
